# A dyadic study of depression, capitalization patterns, and leisure activities in retirement

**DOI:** 10.1093/geroni/igab046.2780

**Published:** 2021-12-17

**Authors:** Ksenya Shulyaev, Dikla Segel-Karpas, Nurit Gur-Yaish

**Affiliations:** 1 Faculty of Social Welfare and Health Science, University of Haifa, Haifa, Hefa, Israel; 2 University of Haifa, Haifa, Hefa, Israel; 3 Oranim Academic College of Education, Haifa, HaZafon, Israel

## Abstract

Late-life relationships, and specifically spousal relations, are increasingly recognized as an important factor shaping the wellbeing, health, social and emotional health of older people. Therefore, a better understanding of the health and well-being trajectories of older adults requires considering the characteristics of their spouses and couple dynamics. This study focused on the actual problem of engagement of recently retired older adults in the community and various leisure activities and examined how both older adults' and spouses’ depression level influence their activities. We also consider the quality of relationships in a couple: how a partner generally responds when the participant discloses good news (capitalization). Fifty-three Israeli couples participated in the current study with one member of the couple 60 or older and retired within the last five years. Recently retired spouses rated their engagement in leisure activities, both spouses reported their level of depression, and partners of retired persons completed the Perceived Responses to Capitalization Attempts Scale. Results show that depression level of recently retired spouses had a direct negative effect (b(SE)=-7.8(3.38), CI(-14.65,-1.04), p=0.02) on their engagement in leisure activities, while the level of their partners' depression had no significant direct effect on retired persons' leisure activities. However, partners’ depression associated (p=0.001) with negative capitalization patterns and mediation analysis showed an indirect effect of partners’ depression via the capitalization (b(SE)=-2.77(1.7), CI(-6.41,-0.04), p=0.03). These results indicate that in encouraging newly retired people to participate in leisure activities it is important to consider both spouses' depression level and capitalization patterns in the couple.

